# Dysregulation of Blood-Brain Barrier and Exacerbated Inflammatory Response in Cx47-Deficient Mice after Induction of EAE

**DOI:** 10.3390/ph14070621

**Published:** 2021-06-28

**Authors:** Filippos Stavropoulos, Elena Georgiou, Irene Sargiannidou, Kleopas A. Kleopa

**Affiliations:** 1 Neuroscience Department, The Cyprus Institute of Neurology and Genetics, Nicosia 2371, Cyprus; filipposs@cing.ac.cy (F.S.); elenag@cing.ac.cy (E.G.); irenes@cing.ac.cy (I.S.); 2 Center for Multiple Sclerosis and Related Disorders, The Cyprus Institute of Neurology and Genetics and Cyprus School of Molecular Medicine, Nicosia 2371, Cyprus

**Keywords:** experimental autoimmune encephalomyelitis, multiple sclerosis, connexins, oligodendrocytes, astrocytes, cytokines, glio-vascular interface

## Abstract

Induction of experimental autoimmune encephalomyelitis (EAE), an animal model of multiple sclerosis (MS), in connexin 32 (Cx32) or Cx47 knockout (KO) mice with deficiency in oligodendrocyte gap junctions (GJs) results in a more severe disease course. In particular, Cx47 KO EAE mice experience an earlier EAE onset and more pronounced disease severity, accompanied by dysregulated pro-inflammatory responses preceding the disease manifestations. In this study, analysis of relevant pro-inflammatory cytokines in wild type EAE, Cx32 KO EAE, and Cx47 KO EAE mice revealed altered expression of *Vcam-1* preceding EAE [7 days post injection (dpi)], of *Ccl2* at the onset of EAE (12 dpi), and of *Gm-csf* at the peak of EAE (24 dpi) in Cx47 KO EAE mice. Moreover, Cx47 KO EAE mice exhibited more severe blood-spinal cord barrier (BSCB) disruption, enhanced astrogliosis with defects in tight junction formation at the glia limitans, and increased T-cell infiltration prior to disease onset. Thus, Cx47 deficiency appears to cause dysregulation of the inflammatory profile and BSCB integrity, promoting early astrocyte responses in Cx47 KO EAE mice that lead to a more severe EAE outcome. Further investigation into the role of oligodendrocytic Cx47 in EAE and multiple sclerosis pathology is warranted.

## 1. Introduction

Multiple sclerosis (MS) is an acquired, autoimmune, and demyelinating disease of the central nervous system (CNS) with a reported prevalence of more than 2.2 million individuals worldwide from 1990 to 2016 [1]. To study MS, various animal models have been developed, among which experimental autoimmune encephalomyelitis (EAE) has been the most extensively studied [2,3]. EAE recapitulates the main aspects of MS, including inflammation, activation of astrocytes and microglia, demyelination, and axonal loss [4,5,6]. Even though the cause of the disease has not yet been fully elucidated, increased permeability of the blood–brain barrier (BBB) and peripheral immune cell infiltration into the CNS are the earliest aberrations observed in brains of MS patients, and are also the early events implicated in EAE development [7,8].

CNS glia cells, including oligodendrocytes, astrocytes, and microglia, are important for maintaining homeostasis and are directly involved in the pathogenesis of MS. Crosstalk between astrocytes and oligodendrocytes is achieved via gap junctions (GJs), which are specialized membrane structures whose building blocks are connexins (Cxs). GJs facilitate the intercellular coupling between oligodendrocytes (oligodendrocyte:oligodendrocyte, O:O), astrocytes (astrocyte:astrocyte, A:A), or oligodendrocytes and astrocytes (oligodendrocyte:astrocyte, O:A). In humans and mice, expression of Cx47 in oligodendrocytes predominates over Cx32, mediating the majority of O:O coupling via homotypic Cx47:Cx47 GJs, and O:A coupling via heterotypic Cx47:Cx43 GJs [9,10,11,12]. Cx32 can also participate in O:O and O:A coupling via homotypic Cx32:Cx32 and heterotypic Cx32:Cx30 GJs, respectively [12,13], but mostly forms intracellular, reflexive, homotypic GJs with itself in oligodendrocytes and the myelin sheath [11,12].

The importance of Cx32 and Cx47 in oligodendrocyte physiology is highlighted by the range of myelin-related disorders reported in humans caused by mutations in their genes [14], and by their altered expression in post-mortem MS brains [15]. Mice lacking either Cx32 or Cx47 do not display evident behavioral defects or CNS demyelination [16,17]; however, when subjected to the inflammatory conditions of EAE they demonstrate more severe CNS pathology than their wild type (WT) counterparts [18]. In particular, Cx47 knockout (KO) mice are more vulnerable to EAE development and progression [18].

A comparative analysis of Cx47 KO, Cx32 KO, and WT EAE mice at 7 days and 12 days post injection (dpi), revealed altered cytokine expression in the connexin-deficient mice, with Cx47 KO EAE mice displaying the greatest alterations. Even before the behavioral manifestation of EAE at 7 dpi, Cx47 KO EAE mice, when compared to WT EAE mice, exhibited a greater alteration in vascular cell adhesion molecule-1 (VCAM-1), C-C motif ligand 2/monocyte chemoattractant protein-1 (CCL2/MCP-1), granulocyte/macrophage-colony-stimulating factor (GM-CSF/CSF2), and granulocyte-colony-stimulating factor (G-CSF/CSF3) protein levels [18].

To further study how *Vcam-1* and the *Ccl2*, *Gm-csf*, and *G-csf* cytokines are associated with EAE disease development, their transcription and translation status were examined in Cx47 KO and Cx32 KO compared to WT mice after EAE induction at pre-onset (7 dpi), onset (12 dpi), and at the peak of the disease (24 dpi). Focusing on the early events responsible for the exacerbated EAE disease in connexin-deficient mice, especially in Cx47 KO mice, we unveiled a dysregulated immune response associated with an earlier disruption of the blood-spinal cord barrier (BSCB) and exacerbated spinal cord infiltration by peripheral immune cells as early as 7 dpi, underscoring the regulatory role of oligodendrocyte GJs in brain homeostasis.

## 2. Results

### 2.1. Cx47 KO EAE Mice Experience an Earlier EAE Onset and a More Severe EAE Disease Course

**Figure 1:**
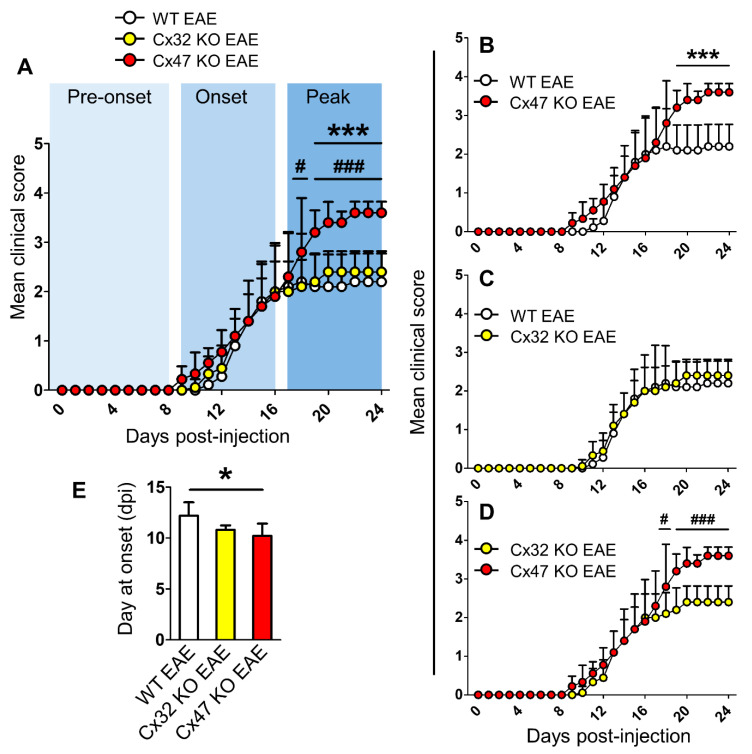
Timeline of clinical course in WT, Cx32 KO, and Cx47 KO EAE mice up to 24 dpi. (**A**–**D**) Comparison of mean clinical scores (MCS) of WT, Cx32 KO, and Cx47 KO EAE mice (**A**), of WT vs. Cx47 KO EAE mice (**B**), of WT vs. Cx32 KO EAE mice (**C**), and of Cx32 KO vs. Cx47 KO EAE mice (**D**). As mice were sacrificed at 7 days, 12 days, and 24 days post-injection (dpi) for subsequent analyses, the sample size used from each group was as follows: 0–7 dpi (*n* = 13 per genotype), 8–12 dpi (*n* = 9 per genotype), and 13–24 dpi (*n* = 5 per genotype). Data are presented as MCS ± SD. Two-way ANOVA with post hoc Bonferroni test: WT EAE vs. Cx47 KO EAE (*** *p* < 0.001); Cx32 KO EAE vs. Cx47 KO EAE (^#^ *p* < 0.05, ^###^ *p* < 0.001). (**E**) Comparison of mean EAE disease onset between *n* = 5 WT, *n* = 5 Cx32 KO, and *n* = 9 Cx47 KO EAE mice. Data is shown as mean ± SD. One-way ANOVA with post hoc Tukey’s HSD test: * *p* < 0.05.

For the purpose of this study a new number of EAE mice was generated similarly to previously published work [18]. EAE was induced in 6–8-week-old WT, Cx32 KO, and Cx47 KO female mice by injection of the MOG_35–55_ peptide. Cx47 KO EAE mice displayed the most severe disease course, especially during the peak phase of the disease (Figure 1A). During the peak of EAE (17–24 dpi, *n* = 5 mice/genotype), the mean clinical score (MCS) of Cx47 KO EAE mice (3.47 ± 0.16) was significantly higher (*p* < 0.001) than that of WT EAE mice (2.15 ± 0.05) from 19–24 dpi (Figure 1B). Comparison between the disease courses of Cx32 KO EAE and WT EAE mice did not reveal any significant difference (Figure 1C). Again, at EAE disease peak, the MCS of Cx47 KO EAE mice was also significantly higher and from that of Cx32 KO EAE mice at 18 dpi (2.8 ± 1.1 in Cx47 KO EAE vs. 2.1 ± 0.55 in Cx32 KO EAE group, *p* < 0.05), and at 19–24 dpi (3.47 ± 0.16 in Cx47 KO EAE vs. 2.37 ± 0.08 in Cx32 KO EAE group, *p* < 0.001) (Figure 1D). Cx47 KO EAE mice (10.22 ± 1.2 dpi, *n* = 9) also had a significantly earlier EAE onset than WT EAE mice (12.2 ± 1.3 dpi, *n* = 5, *p* < 0.05), as they manifested signs of the disease earlier than their WT counterparts (Figure 1E). Cx32 KO EAE mice (10.8 ± 0.45 dpi, *n* = 5, *p* > 0.05) showed a trend towards an earlier EAE onset than WT EAE mice; however, it did not reach statistical significance (Figure 1E).

### 2.2. Cx47 KO EAE Mice Display a Trend of Increased Vcam-1 Translation at 7 dpi

To analyze the mRNA and protein levels of *Ccl2*, *Gm-csf*, *G-csf*, and *Vcam-1* we examined the lumbar spinal cord segment of WT EAE, Cx32 KO EAE, and Cx47 KO EAE mice at 7 dpi, 12 dpi, and 24 dpi. This specific region was selected as EAE lesions predominantly populate the lumbosacral region of the spinal cord [19], reflected in the caudal to rostral pattern of ascending flaccid paralysis observed in EAE mice. The reason for choosing 7 dpi as the first assessment timepoint of cytokines was to check their expression profile before the appearance of the first disease signs. The mRNA and protein levels from the Cx32 KO and Cx47 KO EAE groups were normalized to the WT EAE group, and hence all WT EAE group values were set to 1.

**Figure 2:**
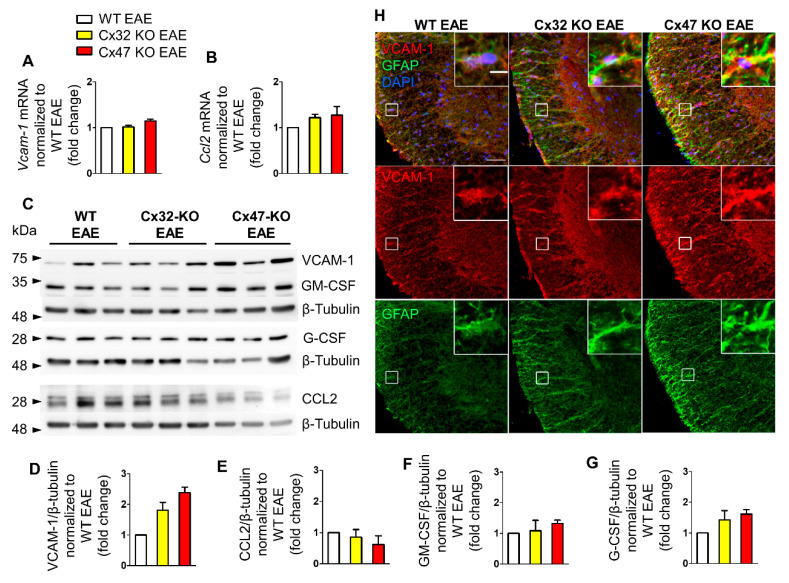
Expression of inflammatory cytokines in the lumbar spinal cord of Cx47 KO and Cx32 KO compared to WT EAE mice at 7 dpi. (**A**,**B**) Assessment of *Vcam-1* (**A**) and *Ccl2* (**B**) relative mRNA transcript levels via RT-qPCR in the lumbar spinal cord of *n* = 4 WT EAE, *n* = 4 Cx32 KO EAE, and *n* = 3 Cx47 KO EAE mice (all with a clinical score of 0) at 7 dpi. *Gapdh* was used as an internal control. Data are presented as mean ± SD. (**C**–**G**): Representative immunoblot (**C**) of lumbar spinal cord tissue lysates from *n* = 3 WT EAE, Cx32 KO EAE, and Cx47 KO EAE mice (all with a clinical score of 0) at 7 dpi with quantification of relative protein levels of VCAM-1 (**D**), CCL2 (**E**), GM-CSF (**F**), and G-CSF (**G**). β-Tubulin was used as loading control. Data are presented as mean ± SD. One-way ANOVA with post hoc Tukey’s HSD test was used for all comparisons. (**H**) Representative immunofluorescence images of the anterolateral lumbar spinal cord region from cross sections obtained from 7 dpi WT EAE and Cx47 KO EAE mice stained with antibodies against VCAM-1 (red), and the astrocytic marker GFAP (green). Nuclei are counterstained with DAPI (blue). Single channels and overlap images are shown as indicated. Magnified views from squared boxes are depicted in insets. Scale bars: in overview 50 μm; in insets 10 μm.

Starting at 7 dpi, when none of the groups manifested signs of the disease, assessment of *Vcam-1* and *Ccl2* mRNA levels did not reveal any obvious difference between the three genotypes. Specifically, *Vcam-1* mRNA levels of Cx32 KO EAE (1.02 ± 0.04-fold change, *n* = 4 mice) and Cx47 KO EAE mice (1.14 ± 0.04-fold change, *n* = 3 mice) were near the WT EAE mice levels (*n* = 4) (Figure 2A). The mRNA levels of *Ccl2* were slightly higher in Cx32 KO (1.22 ± 0.07-fold change, *n* = 4 mice, *p* > 0.05) and Cx47 KO (1.27 ± 0.19-fold change, *n* = 3 mice, *p* > 0.05) EAE compared to WT EAE mice (*n* = 4 mice) (Figure 2B). As we could not find probes successfully detecting *Gm-csf* and *G-csf* mRNA levels, their transcriptional status was not assessed.

Immunoblot analysis (Figure 2C) detected two bands corresponding to CCL2 at ~26 and ~30 kDa, while VCAM-1 was detected at ~74 kDa. The anti-GM-CSF and anti-G-CSF antibodies detected bands at ~34 and 28 kDa, respectively. Quantitative analysis from *n* = 3 mice per genotype revealed a non-significant trend towards an increased VCAM-1 level in the Cx32 KO (1.81 ± 0.26-fold change, *p* > 0.05) and even more in the Cx47 KO EAE group (2.3 ± 0.17-fold change, *p* > 0.05) compared to the WT EAE group (Figure 2D). CCL2 expression was slightly reduced in Cx32 KO (0.86 ± 0.24-fold change, *p* > 0.05), and even more in Cx47 KO EAE mice (0.62 ± 0.28-fold change, *p* > 0.05) without reaching significance (Figure 2E). The GM-CSF level in the Cx32 KO EAE group (1.09 ± 0.33-fold change, *p* > 0.05) did not differ from control level, while it was non-significantly more elevated in the Cx47 KO EAE group (1.31 ± 0.12-fold change, *p* > 0.05) (Figure 2F). G-CSF was more elevated than GM-CSF in both groups, although it did not reach significance, and showed the biggest increase in the Cx47 KO EAE (1.61 ± 0.15-fold change, *p* > 0.05) and a smaller increase in the Cx32 KO EAE group (1.42 ± 0.3-fold change, *p* > 0.05) compared to WT EAE (Figure 2G).

Although the higher expression of VCAM-1 in Cx47 KO EAE mice than in WT EAE controls was not statistically significant, but with a considerable fold change value, its distribution in the spinal cord was further examined. For this reason, lumbar spinal cord sections from the three EAE genotypes were stained for VCAM-1 and the astrocytic marker glial fibrillary acidic protein (GFAP) (Figure 2H). VCAM-1 colocalized with GFAP indicating expression by astrocytes, and its fluorescence intensity appeared more robust in Cx47 KO EAE mice compared to WT EAE controls and Cx32 KO mice, suggesting a tendency for higher VCAM-1 expression in Cx47 KO EAE at 7 dpi.

### 2.3. Increased Ccl2 Transcription in Cx47 KO EAE Mice at 12 dpi

**Figure 3:**
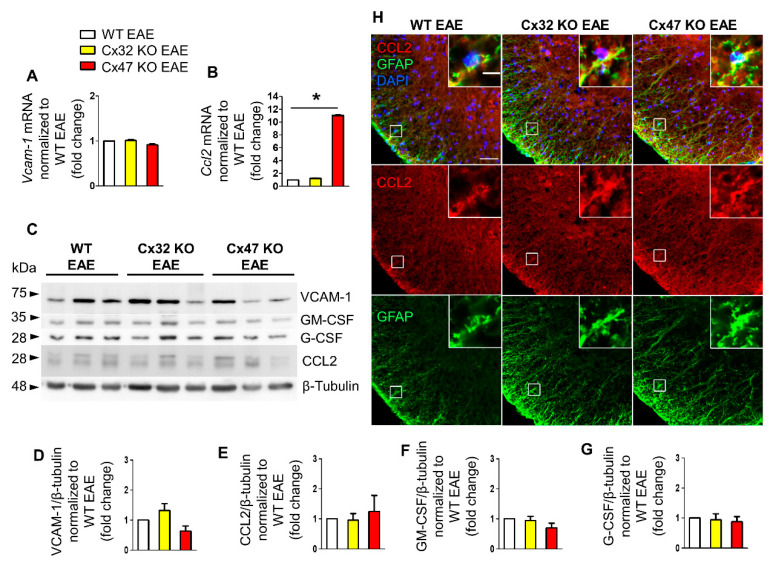
Expression of inflammatory cytokines in the lumbar spinal cord of Cx47 KO and Cx32 KO compared to WT EAE mice at 12 dpi. (**A**,**B**) Assessment of *Vcam-1* (**A**) and *Ccl2* (**B**) relative mRNA transcript levels via RT-qPCR in the lumbar spinal cord of *n* = 4 WT EAE (clinical scores: 0, 0.5, 0, 0), *n* = 4 Cx32 KO EAE (all with a clinical score of 0), and *n* = 4 Cx47 KO EAE mice (all with a clinical score of 0.5) at 12 dpi. *Gapdh* was used as an internal control. Data are presented as mean ± SD. One-way ANOVA with post hoc Tukey’s HSD test was used for all comparisons; * *p <* 0.05. (**C**–**G**) Representative immunoblot (**C**) loaded with lumbar spinal cord tissue lysates from *n* = 3 WT EAE (clinical scores: 0, 0.5, 0), Cx32 KO EAE (clinical scores: 0, 0.5, 0), and Cx47 KO EAE mice (clinical scores: 0.5, 0.5, 0) at 12 dpi and quantification of relative protein levels of VCAM-1 (**D**), CCL2 (**E**), GM-CSF (**F**), and G-CSF (**G**) determined by densitometric analysis using ImageJ. *β*-Tubulin was used as loading control. Data are presented as mean ± SD. (**H**) Representative immunofluorescence images of the anterolateral lumbar spinal cord region from cross sections obtained from 12 dpi WT EAE, Cx32 KO EAE, and Cx47 KO EAE mice (all with a clinical score of 0.5) stained with antibodies against CCL2 (red), and the astrocytic marker GFAP (green). Nuclei are counterstained with DAPI (blue). Single channels and overlap images are shown as indicated with magnified views in insets. Scale bars: in overview 50 μm; in insets 10 μm.

At 12 dpi, EAE mice start to exhibit signs of the disease and their clinical score begins to deviate from zero. During that time, *Vcam-1* mRNA level did not differ between the three genotypes (Figure 3A). Specifically, *Vcam-1* mRNA level reached 1.01 ± 0.02-fold change in Cx32 KO EAE mice (*n* = 4, *p* > 0.05) and 0.91 ± 0.02-fold change (*n* = 4, *p* > 0.05) in Cx47 KO EAE mice compared to WT EAE (*n* = 4 mice). In contrast, *Ccl2* mRNA level was significantly increased (*p* < 0.05) in the Cx47 KO EAE group (11.03 ± 0.07-fold change, *n* = 4 mice) but only minimally elevated (*p* > 0.05) in Cx32 KO EAE group (1.22 ± 0.07-fold change, *n* = 4) compared to WT EAE control group (*n* = 4) (Figure 3B).

Immunoblot analysis (Figure 3C) and quantification showed a non-significant increase of VCAM-1 in the Cx32 KO (1.32 ± 0.23-fold change, *n* = 3 mice) and a non-significant decrease in the Cx47 KO (0.64 ± 0.17-fold change, *n* = 3 mice) compared to WT EAE group (*p* > 0.05) (Figure 3D). CCL2 protein level in the Cx32 KO (0.95 ± 0.23-fold change, *n* = 3 mice, *p* > 0.05) was near the WT level, but appeared slightly elevated in the Cx47 KO EAE group (1.24 ± 0.54-fold change, *n* = 3 mice, *p* > 0.05) (Figure 3E). GM-CSF expression in the Cx32 KO (0.94 ± 0.14-fold change, *n* = 3 mice, *p* > 0.05) did not differ from the WT EAE group but was non-significantly lower in the Cx47 KO EAE (0.69 ± 0.16-fold change, *n* = 3 mice, *p* > 0.05) (Figure 3F). Finally, the expression of G-CSF was not significantly different in either Cx32 KO (0.94 ± 0.2-fold change, *n* = 3 mice, *p* > 0.05) or Cx47 KO (0.88 ± 0.17-fold change, *n* = 3 mice, *p* > 0.05) groups when compared to the WT EAE group (Figure 3G).

Due to the significantly higher transcription of *Ccl2* in Cx47 KO EAE mice, although not supported by its translation analysis, spinal cord sections were stained for CCL2 and GFAP (Figure 3H). Among the three different genotypes, a higher CCL2 fluorescent intensity was observed in the Cx47 KO EAE group. Expression of CCL2 by astrocytes was verified via CCL2 and GFAP co-localization.

### 2.4. Increased Gm-Csf Translation in Cx47 KO EAE Mice at 24 dpi

**Figure 4:**
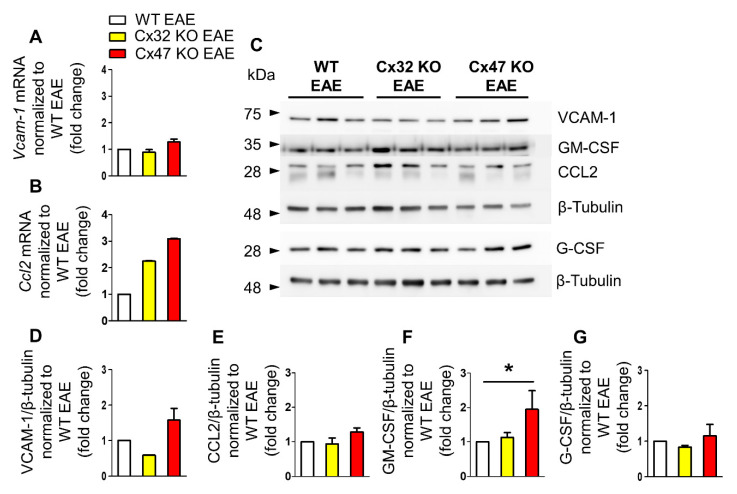
Expression of inflammatory cytokines in the lumbar spinal cord of Cx47 KO and Cx32 KO compared to WT EAE mice at 24 dpi. (**A**,**B**) Assessment of *Vcam-1* (**A**), and *Ccl2* (**B**) relative mRNA transcript levels via RT-qPCR in the lumbar spinal cord of *n* = 4 WT EAE (clinical scores: 2, 2, 3, 2.5), *n* = 4 Cx32 KO EAE (clinical scores: 2.5, 2, 2, 3), and *n* = 4 Cx47 KO EAE (clinical scores: 3.5, 4, 3.5, 3.5) at 24 dpi. *Gapdh* was used as an internal control. Data are presented as mean ± SD. (**C**–**G**) Representative immunoblot of lumbar spinal cord tissue lysates from *n* = 3 WT EAE (clinical scores: 3.5, 3, 1.5), Cx32 KO EAE (clinical scores: 4, 4.5, 2.5), and Cx47 KO EAE mice (clinical scores: 4, 3.5, 4) at 24 dpi and quantification of relative protein levels of VCAM-1 (**D**), CCL2 (**E**), GM-CSF (**F**), and G-CSF (**G**) determined by densitometric analysis using ImageJ. *β*-Tubulin was used as loading control. Data are presented as mean ± SD. One-way ANOVA with post hoc Tukey’s HSD test: * *p* < 0.05.

During the peak of EAE disease at 24 dpi, the mean clinical score of EAE mice is the highest as mice exhibit severe signs of the disease. At that time, *Vcam-1* transcription showed a minor reduction in the Cx32 KO EAE (0.89 ± 0.1-fold change, *n* = 4 mice, *p* > 0.05) and a marginal increase in the Cx47 KO EAE (1.27 ± 0.11-fold change, *n* = 4 mice, *p* > 0.05) group compared to WT EAE (*n* = 4 mice) group, without reaching statistical significance (Figure 4A). *Ccl2* transcription, on the other hand, showed an elevated trend in both mutants compared to the WT EAE group. Cx32 KO mice showed a 2.25 ± 0.02-fold change (*n* = 4 mice, *p* > 0.05) and Cx47 KO EAE a 3.09 ± 0.02-fold change (*n* = 4 mice, *p* > 0.05) of *Ccl2* mRNA levels (Figure 4B).

Expression analysis at 24 dpi revealed a significant increase of GM-CSF protein level in the Cx47 KO EAE group (*n* = 3 mice) compared to both the Cx32 KO EAE (*n* = 3 mice) and WT EAE groups (*n* = 3 mice) (Figure 4D–G). VCAM-1 displayed a tendency towards a reduced protein level in the Cx32 KO group (0.59 ± 0.01-fold change, *p* > 0.05) and towards an increased level in the Cx47 KO group (1.57 ± 0.34-fold change, *p* > 0.05) in comparison to the WT EAE group (Figure 4D). The CCL2 level in the Cx32 KO EAE group (0.93 ± 0.17-fold change, *p* > 0.05) did not differ from control group level, while in the Cx47 KO (1.28 ± 0.12-fold change, *p* > 0.05) a non-significant marginal increase was detected (Figure 4E). The level of GM-CSF in the Cx32 KO group (1.13 ± 0.14-fold change, *p* > 0.05) was near control group level, but was significantly higher (*p* < 0.05) in the Cx47 KO EAE group (1.95 ± 0.54-fold change) (Figure 4F). G-CSF expression in Cx32 KO EAE (0.83 ± 0.05-fold change, *p* > 0.05) and in Cx47 KO EAE mice (1.15 ± 0.33-fold change, *p* > 0.05) was near control group expression level (Figure 4G).

### 2.5. T-Cell Infiltration Is Higher in the Spinal Cords of 7 dpi Cx47 KO EAE Mice

**Figure 5:**
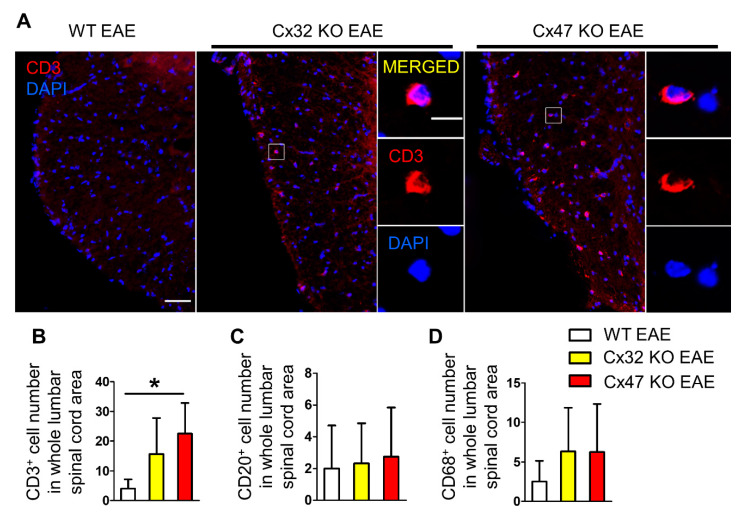
Assessment of CD3^+^ T-cell, CD20+ B-cell, and CD68+ macrophage infiltrates in the lumbar spinal cord of WT and Cx47 KO EAE mice at 7 dpi. (**A**) Representative immunofluorescence overview images of lumbar spinal cord cross sections from 7 dpi WT EAE, Cx32 KO EAE, and Cx47 KO EAE mice as indicated (all with a clinical score of 0), stained against the T-cell marker CD3 (red), and counterstained with DAPI (blue). Higher magnification single channels and overlap images from squared boxes are shown in insets on the right. Scale bars: in overviews = 50 μm; in insets = 10 μm. Counts of CD3^+^ T-cells (**B**), CD20+ B-cells (**C**), and CD68+ monocytes/macrophages (**D**) in the whole lumbar spinal cord area of *n* = 4 WT EAE, *n* = 3 Cx32 KO EAE, and *n* = 4 Cx47 KO EAE mice. Data are presented as mean ± SD. One-way ANOVA with post hoc Tukey’s HSD test: * *p* < 0.05.

In order to identify the factors driving the earlier EAE onset in mice lacking Cx47, their spinal cords were examined for infiltration of peripheral immune cells, as inflammatory-cell infiltrates have been reported in spinal cords of EAE mice prior to disease onset [20]. Lumbar spinal cord sections from WT, Cx32 KO, and Cx47 KO EAE mice were stained with CD3 (T-cell marker), CD20 (B-cell marker), and CD68 (monocyte/macrophage marker) at disease pre-onset (7 dpi) (Figure 5A). A non-significant increase in CD3^+^ cell infiltrates was spotted in the Cx32 KO EAE group (15.67 ± 12.1 cells, *n* = 3 mice, *p* > 0.05), but their number was significantly greater in the Cx47 KO (22.5 ± 10.34 cells, *p* < 0.05, *n* = 4 mice) than in the WT EAE group (4 ± 3.16 cells, *n* = 4 mice) (Figure 5B). Most CD3^+^ cell infiltrates were located in the white matter and close to the meninges. When assessing the number of CD20^+^ cell infiltrates, no obvious difference was found between the WT (2 ± 2.71 cells, *n* = 4 mice), Cx32 KO (2.33 ± 2.52 cells, *n* = 3 mice, *p* > 0.05), and Cx47 KO EAE group (2.75 ± 3.1 cells, *n* = 4 mice, *p* > 0.05) (Figure 5C). Regarding CD68^+^ cell infiltrates, these were non-significantly increased (*p* > 0.05) in the Cx32 KO (6.33 ± 5.51 cells, *n* = 3 mice) and Cx47 KO (6.25 ± 6.08 cells, *n* = 4 mice) in comparison to the WT EAE group (2.5 ± 2.65 cells, *n* = 4 mice) (Figure 5D).

### 2.6. More Severe BSCB and Glia Limitans Disruption in 7 dpi Cx47 KO EAE Mice

Since spinal cords from Cx47 KO EAE mice were characterized by a significantly greater number of CD3^+^ T-cell infiltrates, we assessed the integrity of their BSCB as a more disrupted BSCB would facilitate the earlier entry of peripheral immune cells into their CNS. Additionally, the increased number of cell infiltrates could further compromise BSCB integrity. Considering the higher burden of CD3^+^ T-cell infiltrates in Cx47 KO EAE mice and together with their more severe EAE disease course than Cx32 KO mice, for the following analyses only specimens from the Cx47 KO and WT EAE control groups were included.

**Figure 6:**
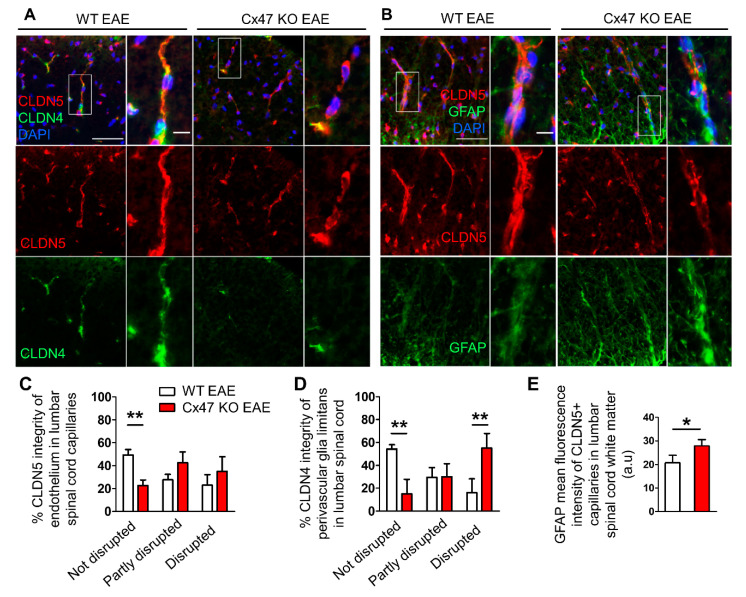
Increased disruption of endothelial and astrocytic tight junctions and increased reactivity of perivascular glia limitans in the lumbar spinal cord of 7 dpi Cx47 KO EAE mice. (**A**,**B**) Representative immunofluorescence images of lumbar spinal cord cross sections from 7 dpi WT EAE and Cx47 KO EAE mice as indicated (both with a clinical score of 0), stained with antibodies against the endothelial tight junction (TJ) protein CLDN5 (red) and the astrocytic TJ protein CLDN4 (green) in **A**, or against CLDN5 (red) and the astrocytic marker GFAP (green) in **B**. Nuclei are counterstained with DAPI (blue). Separate channels and overlays are shown. Magnified views from rectangular boxes are depicted on the right (Scale bars: in overviews 50 μm, in insets: 10 μm). Magnified view from WT EAE mouse (**A**) depicts a vessel with an intact CLDN5 and CLDN4 pattern, while a vessel from Cx47 KO EAE (**A**) shows a partly disrupted CLDN5 and a completely disrupted CLDN4 pattern. (**C**,**D**) Semi-quantitative scoring of endothelial CLDN5 (**C**) and of perivascular CLDN4 (**D**) staining pattern in lumbar spinal cord capillaries of WT EAE (*n* = 3) and Cx47 KO EAE (*n* = 4) mice. Data are presented as mean ± SD. Two-way ANOVA with post hoc Bonferroni test: ** *p* < 0.01. (**E**) Semi-quantitative analysis of GFAP fluorescence intensity, overlapping with vessel-like CLDN5 fluorescence pattern (*n* = 10 vessels/mouse), using ImageJ from the lumbar spinal cord white matter of *n* = 4 WT EAE mice and *n* = 4 Cx47 KO EAE mice (clinical score of all mice was 0). a.u., arbitrary units. Data are presented as mean ± SD. Unpaired student’s *t*-test: * *p* < 0.05.

For the assessment of BSCB integrity, lumbar spinal cord sections were stained for the tight junction (TJ) protein components Claudin 4 (CLDN4) and 5 (CLDN5), which regulate the permeability of the BBB/BSCB (Figure 6A) [21,22,23]. Infiltrating cells need to overcome two barriers at the glio-vascular interface in order to get access to the CNS. First, they need to overcome the endothelial barrier that in addition to other TJ proteins also normally expresses CLDN5. The second barrier is the glia limitans barrier, formed by the astrocytic endfeet processes, which among other TJ proteins, expresses CLDN4 under inflammatory conditions [23,24].

The use of a semi-quantitative scoring system revealed that the percentage of intact spinal cord white matter capillaries showing a continuous, non-disrupted CLDN5 staining pattern was significantly lower (*p* < 0.01) in the Cx47 KO (22.5 ± 5%, *n* = 4 mice) than in WT EAE controls (51.92 ± 4.73%, *n* = 3 mice) (Figure 6B). The percentage of capillaries with partly disrupted BSCB, classified by their partly interrupted CLDN5 staining pattern, tended to be greater in the Cx47 KO (42.5 ± 9.57%, *n* = 4 mice, *p* > 0.05) than in the WT EAE group (30.38 ± 4.73%, *n* = 4 mice) (Figure 6B). Likewise, the percentage of capillaries with completely disrupted BSCB, evident in their reduced CLDN5 fluorescence intensity and the interrupted staining pattern along their axes, tended to be higher in the spinal cords of Cx47 KO (35 ± 12.91%, *n* = 4 mice, *p* > 0.05) than in WT EAE controls (17.69 ± 9.32%, *n* = 3 mice), but this difference did not achieve statistical significance (Figure 6B).

Semi-quantitative scoring of CLDN4, showed a significantly lower percentage of capillaries ensheathed by a non-disrupted glia limitans in Cx47 KO (15 ± 12.91%, *n* = 4 mice) than in WT EAE mice (53.57 ± 5.05%, *n* = 3 mice, *p* < 0.01) as assessed by the continuous, non-disrupted CLDN4 staining pattern (Figure 6C). The percentage of capillaries with a partially disrupted glia limitans, as assayed by the non-continuous CLDN4 staining pattern, in the Cx47 KO group (30 ± 11.55%, *n* = 4 mice, *p* > 0.05) did not differ from the control group (27.86 ± 11.11%, *n* = 3 mice). Finally, the percentage of capillaries with a completely disrupted glia limitans, evaluated by their near absent CLDN4 staining around capillaries, was significantly higher (*p* < 0.01) in Cx47 KO (55 ± 12.91%, *n* = 4) than in WT EAE mice (18.57 ± 16.16%, *n* = 3 mice) (Figure 6C).

Given the exacerbated disruption of glia limitans in Cx47 KO EAE mice as assessed by the abnormal expression of CLDN4 in astrocyte endfeet, we next studied astroglial pathology in lumbar spinal cord sections from WT and Cx47 KO EAE mice at 7 dpi stained with the astrocytic marker GFAP (Figure 6D). Sections were also stained with the endothelial TJ protein CLDN5 to mark vessels (Figure 6D). Specifically, the perivascular glia limitans area was analyzed by measuring the GFAP surface covering parenchymal CLDN5^+^ vessels. Cx47 KO EAE mice exhibited significantly higher (*p* = 0.014) GFAP mean intensity (27.61 ± 3.23 arbitrary units (a.u.), *n* = 4) than WT EAE mice (20.65 ± 3.2 a.u., *n* = 4) (Figure 6E). Thus, a more hypertrophic reactive profile of astrocytic endfeet at the glia limitans is observed in Cx47 KO EAE mice before disease onset, while the formation of TJs by CLDN4 is more severely disrupted as shown above.

### 2.7. Cx47 KO EAE Mice Experience a Non-Significant Reduction in Cx43 at 7 dpi

**Figure 7:**
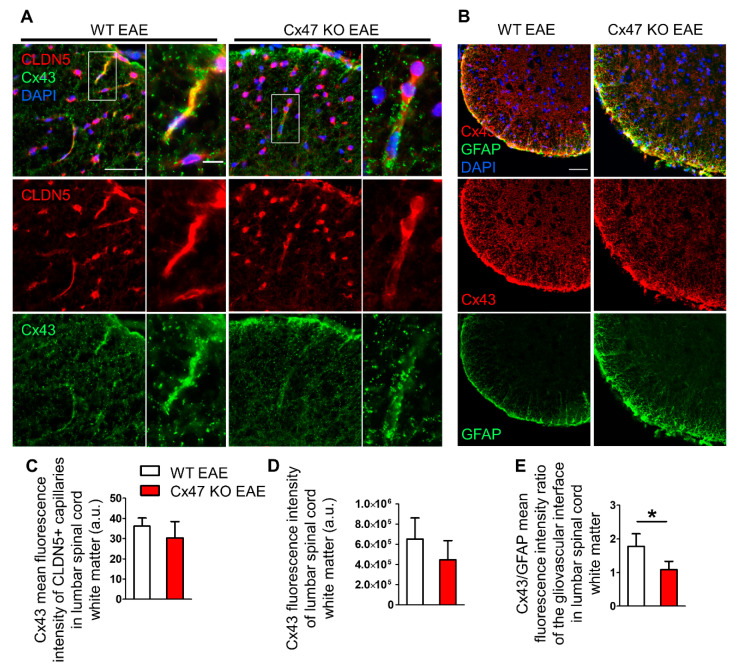
Cx43 immunoreactivity at the glio-vascular interface and lumbar spinal cord white matter of Cx47 KO and WT EAE mice at 7 dpi. (**A**) Representative immunofluorescence images of lumbar spinal cord cross sections from a WT and Cx47 KO EAE mouse (both with a clinical score of 0) at 7 dpi (Scale bar = 50 μm). Tissues were stained with antibodies against the endothelial TJ protein CLDN5 (red) and Cx43 (green). Nuclei are counterstained with DAPI (blue). Single channels and overlap images are provided. Magnified views from rectangular boxes are depicted on the right (Scale bar = 10 μm). (**B**) Cross sections of the anterolateral lumbar spinal cord region from a WT and Cx47 KO EAE mouse (both with a clinical score of 0) at 7 dpi immunostained for Cx43 (red) and GFAP (green) show reduction of Cx43 in the Cx47 KO (Scale bar = 50 μm). (**C**) Semi-quantitative analysis of (**A**), where mean intensity of Cx43 fluorescence overlapping with the vessel-like CLDN5 fluorescence pattern (*n* = 9–11 vessels/mouse) was assayed with ImageJ from the lumbar spinal cords of *n* = 4 WT EAE mice and *n* = 4 Cx47 KO EAE mice. (**D**) Semi-quantitative analysis of (**B**), where six 4 cm × 4 cm regions of interest (ROI) from the anterolateral white matter region were drawn from one image per WT EAE (*n* = 4, clinical score of all mice was 0) and per Cx47 KO EAE mouse (*n* = 4, clinical score of all mice was 0). (**E**) Perivascular Cx43 mean fluorescence intensity from each mouse was divided by their respective perivascular GFAP mean fluorescence intensity. a.u., arbitrary units. Data are presented as mean ± SD. Unpaired student’s *t*-test was used for all comparisons: * *p* < 0.05. Scale bar = 50 μm; Magnified view scale bar = 10 μm.

Astrocytes are extensively coupled via GJs mainly composed of Cx43, and such GJ connectivity is also present at the glia limitans, interconnecting astrocytic endfeet [25,26,27]. Double staining of lumbar spinal cord for Cx43 and the endothelial TJ component CLDN5 at 7 dpi (Figure 7A), revealed a non-significant decrease in Cx43 fluorescence intensity at the perivascular glia limitans of Cx47 KO EAE mice (30.38 ± 8.05 a.u, *n* = 4, *p* = 0.25) compared to WT EAE (36.14 ± 4.03 a.u., *n* = 4) (Figure 7C). The overall Cx43 fluorescence intensity (Figure 7B) in the anterolateral white matter of Cx47 KO (4.45 × 10^5^ ± 1.91 × 10^5^ a.u, *n* = 4), was also diminished compared to WT EAE mice (6.52 × 10^5^ ± 2.08 × 10^5^ a.u, *n* = 4, *p* = 0.19); however, it did not reach statistical significance (Figure 7D).

Due to the tendency for reduction of total Cx43 in Cx47 KO EAE mice together with the prominent hypertrophy of astrocytic processes forming the glia limitans, we also evaluated the Cx43 to GFAP ratio at the glia limitans. For this purpose, the mean fluorescence intensity of Cx43 and GFAP at the glia limitans from the same mice were utilized. The Cx43 to GFAP ratio was significantly reduced in Cx47 KO (1.09 ± 0.25 a.u, *n* = 4 mice, *p* = 0.021) compared to WT EAE mice (1.82 ± 0.37 a.u, *n* = 4) (Figure 7E). Thus, the exacerbated early BSCB disruption in Cx47 KO EAE mice is characterized by a greatly reduced astrocytic Cx43/GFAP ratio at the glio-vascular interface prior to disease onset, along with the reduction of TJ protein CLDN4 at the glia limitans.

## 3. Discussion

Comparison of the EAE-induced pathological changes on a WT background, and on the background of deficiency for one of the two major oligodendrocyte connexins, revealed important variations in the profile of pro-inflammatory factors, especially in Cx47 KO EAE mice. EAE mice lacking Cx47 showed a higher trend of *Vcam-1* protein expression at 7 dpi, and significantly higher *Ccl2* mRNA level at 12 dpi and *Gm-csf* protein level at 24 dpi. They also exhibited an exacerbated early disruption of their BSCB at 7 dpi, with a more severe impairment of vascular CLDN5-based TJs, and hypertrophic astrocytic glia limitans with disrupted CLDN4-based TJ formation and initiation of Cx43 loss. The exacerbated cytokine and BSCB abnormalities in Cx47 KO EAE mice were associated with increased T-cell infiltration before disease onset.

Cx47 KO EAE mice exhibited a tendency of increased *Vcam-1* translation at disease pre-onset (7 dpi) and at disease peak (24 dpi). VCAM-1 is minimally expressed in the non-inflamed CNS [28,29], while it is upregulated under inflammatory conditions, and is expressed by reactive astrocytes [30], stimulated ECs [29,31], and activated microglia [32]. VCAM-1 binds to the very late antigen-4 (VLA-4)/α4β1-integrin on leukocytes, promoting their adhesion to the vascular endothelium and ultimately their migration into the inflamed tissue [31,33]. Natalizumab used in MS treatment blocks the α4β1/VCAM-1 interaction which is required for EAE development [34], thus preventing the migration of leukocytes through the BBB into the inflamed CNS [31,33]. On the other hand, diminished *Vcam-1* translation in the Cx47 KO EAE group during EAE onset at 12 dpi could result from cell surface VCAM-1 shedding due to proteolytic cleavage by metalloproteinases [35,36]. This could either attenuate immune cell infiltration [36,37], or induce leukocyte chemotaxis to the inflamed tissue [38,39].

A significantly higher *Ccl2* transcription in EAE mice lacking Cx47 was evident during EAE onset (12 dpi). This could be due to the increase in inflammation state associated with the earlier EAE onset in Cx47 KO mice. Although not reaching significance, *Ccl2* translation in the Cx47 KO EAE group displayed a slight increase at the onset and peak (24 dpi) of EAE disease, while it was slightly lower during pre-onset (7 dpi) EAE. CCL2 is a chemotactic cytokine (chemokine) that attracts C-C chemokine receptor type 2 (CCR2)-expressing peripheral immune cells into the CNS during EAE development [40,41]. CCL2 is expressed in the CNS both by ECs and astrocytes [42]. Due to the higher T-cell infiltration in Cx47 KO EAE mice at disease pre-onset, consumption of CCL2 by migrating CCR2-expressing cells at the inter-endothelial junctions and abluminal surface of ECs [42,43] along with low transcription, may contribute to the reduction of CCL2 level.

A discrepancy observed between transcriptional and translational levels of *Vcam-1* at 7 dpi and of *Ccl2* at 12 dpi could be attributed to post-transcriptional modifications such as RNA interference (RNAi), post-translational modifications or proteolysis, which may affect mRNA and/or protein half-life, respectively [35,43,44,45]. Furthermore, due to tissues harvested from different mice for the RT-qPCR and immunoblot analyses per disease time-point, another contributing factor could be differences in EAE severity between mice of the same group.

At disease peak, only GM-CSF was significantly elevated in the Cx47 KO EAE group. GM-CSF has pleiotropic and controversial effects on EAE pathogenesis. GM-CSF promotes MOG_35–55_-specific T-cell priming [46], and secretion from T-cells alone facilitates EAE disease onset, indicating that T-cell secreted and not CNS derived GM-CSF is required for driving EAE pathogenesis [47]. This may explain the slightly higher GM-CSF protein level in Cx47 KO EAE mice at 7 dpi, probably coming from their increased number of T-cell infiltrates. In contrast, another study indicated that GM-CSF signaling does not affect EAE onset but exacerbates tissue damage during disease peak by promoting CNS build-up of peripheral immune cells [46]. Thus, greater GM-CSF expression in Cx47 KO EAE mice at 24 dpi may correlate with their higher burden of infiltrated immune cells and greater EAE severity [18].

G-CSF, like GM-CSF, also has pleiotropic effects on EAE pathogenesis. It can drive the expansion and accumulation of bone marrow derived neutrophils and monocytes in the bloodstream during EAE pre-onset [48], promotes an anti-inflammatory activity by inducing the apoptosis of MOG_35–55_ autoreactive T-cells [49], or have a neuroprotective role [50,51]. In the normal rat brain, G-CSF has a predominantly neuronal expression, while it is also expressed by astrocytes exposed to pro-inflammatory cytokines [52]. As G-CSF can also pass the intact BBB of WT rats [53], the trend towards a relatively higher content in the spinal cords of Cx47 KO EAE mice at 7 dpi could either be from the secretions of activated immune cells in the periphery, passing through their already weakened BBB, or from reactive astrocytes, or both.

The more compromised BSCB of Cx47 KO EAE mice days before disease manifestation, and their more reactive glia limitans formed by astrocytic endfeet, accompanied by increased disruption of CLDN4-based TJs, seem to facilitate the earlier invasion of spinal cord parenchyma by circulating immune cells, especially CD3^+^ T lymphocytes. BBB/BSCB leakage [8,54], along with hypertrophic astrocytes presented with swollen endfeet processes bordering capillaries and increased GFAP immunoreactivity [54,55] have been described prior to cellular infiltration in the CNS. This earlier breakdown of the glio-vascular interface and entry of circulating cells into the CNS of Cx47 KO EAE mice, may explain their earlier onset of neurological signs. Moreover, their higher burden in CD3^+^ T lymphocytes, compared to the other infiltrating immune cell populations at pre-onset EAE, is in concordance with findings supporting EAE to be a T-cell mediated disease [56]. This higher number of CD3^+^ T-cell infiltrates in Cx47 KO EAE mice could be responsible for their more disrupted CLDN4-based TJs, as CD3^+^ T-cells degrade astrocytic CLDN4 [23]. Pericytes also regulate BBB/BSCB integrity [57] and, in the presence of inflammation, these cells have been reported to downregulate their contact sites with ECs, finally destabilizing from them [58,59,60].

During the acute stages of EAE, Cx43 GJs, mostly forming the astrocytic syncytium [25,26], are reduced within lesions and perilesional areas in WT EAE mice [61]. Disruption of Cx43 GJ coupling at early EAE could be in part because of massive peripheral immune cell infiltration [62], subsequently serving as a mechanism to prevent the propagation of apoptotic stimuli between astrocytes [63,64]. Based on the aforementioned, the non-significantly lower Cx43 immunoreactivity in the lumbar spinal cords of Cx47 KO EAE mice at 7 dpi, signifying an initiation of Cx43 loss in these mice, could be a consequence of their higher burden in T-lymphocytes.

**Figure 8:**
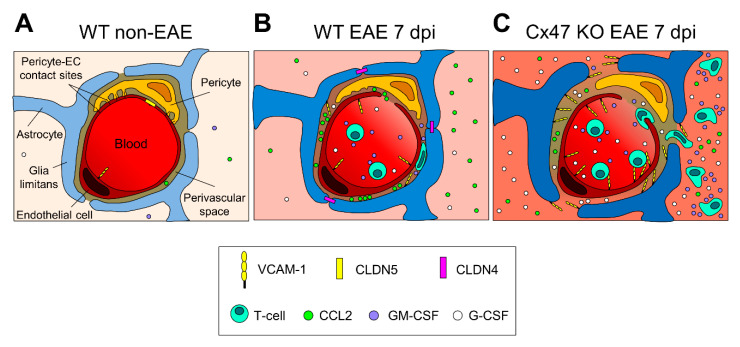
Proposed diagrammatic representation of the glio-vascular interface differences between the 6–8 weeks old WT EAE and Cx47 KO EAE mice at pre-onset EAE. (**A**) In the non-inflamed glio-vascular interface of WT mice, the BBB/BSCB is sealed by endothelial CLDN5-based TJs, glia limitans end-feet are not reactive and do not express CLDN4-based TJs, and pericytes form contact sites with endothelial cells (ECs). CCL2, VCAM-1, GM-CSF, and G-CSF are expressed at low levels. (**B**) In the inflamed glio-vascular interface of WT EAE mice at 7 dpi, endothelial CLDN5-based TJs are downregulated opening the BBB/BSCB, and ECs upregulate VCAM-1 expression, attracting primed T-cells to the perivascular space (PVS). Glia limitans end-feet become reactive and express CLDN4-based TJs sealing the glia limitans barrier, thus impeding access of incoming primed T-cells to the CNS parenchyma. (**C**) In the inflamed glio-vascular interface of Cx47 KO EAE mice at 7 dpi, endothelial CLDN5-based TJs are more downregulated and VCAM-1 is more upregulated than in WT EAE mice (**B**), attracting more primed T-cells from the periphery into the PVS. G-CSF, and GM-CSF are also more upregulated, and glia limitans end-feet are more reactive. At the PVS, primed T-cells cleave CLDN4-based TJs and open the glia limitans barrier, making their entry into the CNS parenchyma where they keep secreting cytokines augmenting the CNS inflammatory process. Under inflammatory conditions (**B** and **C**), stimulated pericytes hypothetically downregulate their contact sites with ECs and detach from them.

During the early EAE stages, a partial disruption of the BBB/BSCB first causes a fluid influx into the CNS, affecting predominantly astrocytes leading to astrogliosis. This is followed by further BBB/BSCB disruption and CNS infiltration by “primed” peripheral immune cells, ultimately leading to loss of Cx43 as shown by this study, and finally demyelination, axonal damage, and loss [54]. These series of events are more pronounced and seem to occur earlier in Cx47 KO EAE mice than in their Cx32 KO EAE and WT EAE counterparts (Figure 8), most probably due to a predisposition of Cx47 KO EAE mice to a weaker BSCB. Comparison of WT and Cx47 KO mice at baseline conditions (not submitted to EAE induction) revealed pathological alterations in the CNS of Cx47 KO mice. These included delayed myelin formation, accompanied by astrogliosis, and microglial activation in 10-day-old Cx47 KO mice brains [65], and sporadic myelin vacuolation (but not demyelination) in optic nerve fibers from 5–14 weeks old Cx47 KO mice [66]. Therefore, even in the absence of EAE, Cx47 KO mice display CNS perturbations that make them more susceptible to EAE development.

If a functionally compromised BSCB is the earliest step in the sequence of EAE events, then how does complete ablation of Cx47 predispose to an exacerbated BSCB disruption? Expression of Cx47 in ECs is excluded since no Cx47 immunoreactivity is spotted on ECs of CNS capillaries [18]. On the other hand, oligodendrocyte precursor cells (OPCs) in vitro support BBB integrity by increasing the expression of the TJ proteins ZO-1, occludin, and CLDN5 via secretion of the multipotent cytokine transforming growth factor β1 (TGF-β1) [67]. Mice with conditional KO (cKO) of *Tgfb1* from OPCs alone, displayed pronounced cerebral hemorrhage with degraded ZO-1 [67]. Moreover, in WT mice, some OPCs were found attached to the basal lamina surrounding ECs, being in close proximity to the CNS endothelium [67]. As OPCs support BBB integrity, and along with oligodendrocytes, also highly express Cx47 [15,68], a complete removal of Cx47 could possibly affect their stabilizing effects on BBB/BSCB, rendering the BSCB of Cx47 KO mice more prone to disruption.

Exacerbated EAE disease outcome was recently reported in oligodendroglia-specific inducible Cx47 cKO (icKO) mice [69], supporting that if there is any contribution of other Cx47-expressing cell population in the CNS apart from oligodendrocytes, such as the CNS lymphatic epithelium [70,71], this has little if any effect on EAE pathogenesis. However, to address this, a direct comparison of EAE in oligodendroglia-specific Cx47-cKO and Cx47 KO mice would be useful.

Our study has several limitations. More studies need to be carried out to examine CLDN4 and CLDN5 expression and translation dynamics between the three EAE genotypes during disease initiation and development. Additionally, the small animal groups used in our evaluation may have limited the statistical significance of several differences found between the genotypes, especially in the assessment of cytokines.

## 4. Materials and Methods

### 4.1. Experimental Animals

C57BL/6N (WT) mice, along with *Gjb1*-null/Cx32 KO and *Gjc2*-null/Cx47 KO mice fully backcrossed into the C57BL/6N background (at least 10 generations), all of them female, were housed in the pathogen-free mouse facility of the Cyprus Institute of Neurology and Genetics under controlled environmental conditions. All animal procedures were approved by the Cyprus Government’s Chief Veterinary Officer according to EU guidelines (License Nr. CY/EXP/PR.L1/2017, Date of approval: 19 June 2017, Expiration date: 18 June 2022). Mice had ad libitum access to standard chow and water and were genotyped prior to use.

### 4.2. EAE Induction and Disease Assessment

EAE was induced by subcutaneous (s.c.) injection at the base of the tail (day 0) with a 200 μL emulsion containing 200 μg MOG_35–55_ peptide (NH2–MEVGWYRSPFSRVVHLYRNGK–COOH) synthesized at Johns Hopkins University lab, Baltimore, MD, USA) dissolved in Complete Freund’s Adjuvant (CFA), as in [72] with minor modifications, as previously described [18]. Briefly, 6–8 weeks old, female mice of WT, Cx32 KO, and Cx47 KO background were injected with the MOG_35–55_ peptide immunogen to induce EAE. The MOG_35–55_ peptide was first dissolved in phosphate-buffered saline (PBS) at 2 mg/mL and mixed at 1:1 ratio with CFA containing 5 mg/mL heat-inactivated *Mycobacterium tuberculosis*. Additionally, mice were also injected intraperitoneally (i.p.) with 200 ng of pertussis toxin (1 ng/μL in PBS) on the same day of peptide immunogen administration (denoted as day 0), and 2 days after the injection of the peptide (at 2 dpi). Disease assessment was performed daily by scoring mice on a scale of 0 to 5 (gradation 0.5) [72]. The scoring system was: 0, no disease signs; 1, loss of tail tone; 2, wobbly gait; 3, hind limb paralysis; 4, moribund; and 5, death.

### 4.3. Tissue Processing and Immunofluorescence Staining

Mice were deeply anesthetized with 2, 2, 2 Tribromoethanol (Avertin), and then, were transcardially perfused with iced-cold 0.9% saline followed by 4% paraformaldehyde (PFA) in 0.1 M phosphate buffer (PB) pH 7.2. Spinal cords were post-fixed in 4% PFA for 1 h and incubated in 20% sucrose solution (in 0.1 M PB, pH 7.2) prior to freezing, to avoid the formation of freezing artifacts in tissues. Spinal cords were divided into cervical, thoracic, and lumbosacral segments, embedded in OCT compound (Scigen Scientific Inc., Gardena, CA, USA), and stored at −80 °C. Spinal cord tissue blocks were cryosectioned (12 μm thick) in a cryotome, mounted on glass slides, and stored at −20 °C.

For the immunofluorescence staining, slides were first permeabilized with cold acetone for 10 min at 20 °C, and incubated in blocking solution, consisting of 0.5% Triton X-100 diluted in 5% bovine serum albumin (BSA), for 1 h at room temperature (RT). Sections were then incubated at 4 °C overnight (O/N) with the desired primary antibody in blocking solution: rabbit anti-CCL2/MCP-1 (Millipore, 1:100), rabbit anti-VCAM-1 (LifeSpan BioSciences, 1:200), mouse anti-GFAP (Invitrogen, 1:300), rabbit anti-G-CSF (Abcam, 1:100), rabbit anti-GM-CSF (Abcam, 1:100), rat anti-CD68:Alexa Fluor 488 (Serotec, 1:50), rabbit anti-CD3 (Invitrogen, 1:100), goat anti-CD20 (Santa Cruz Biotechnology, 1:100), mouse anti-CLDN4 (Santa Cruz Biotechnology, 1:200), rabbit anti-CLDN5 (Abcam, 1:100), mouse anti-Cx43 (Millipore, 1:50), or rabbit anti-Cx43 (Cell Signalling Technology, 1:50). The next day, sections were washed with 1 X PBS, and incubated for 1 h at RT with the respective fluorescent-dye-coupled secondary antibody in blocking solution. The secondary antibodies utilized in this study were the following: anti-rabbit TRITC (Jackson ImmunoResearch, 1:3000), anti-mouse FITC (Jackson ImmunoResearch, 1:1500), anti-mouse Alexa Fluor 488 (Invitrogen, 1:4000), and anti-goat FITC (Jackson ImmunoResearch, 1:700). To visualize the nuclei, sections were counterstained with DAPI (Sigma-Aldrich, St. Louis, MO, USA), and covered with Fluorescence Mounting Medium (Dako Omnis, Agilent Technologies Inc., Santa Clara, CA, USA). Images were taken with a Nikon Eclipse Ni-E microscope with the DS-Fi2 camera head at 20 X and 40 X magnifications using the NIS-Elements imaging software.

### 4.4. Immunoblot Analysis

Lumbar spinal cord segments were lysed in RIPA buffer (10 mM sodium phosphate pH 7.0, 150 mM NaCl, 2 mM EDTA, 50 mM sodium fluoride, 1% NP-40, 1% sodium deoxycholate and 0.1% sodium dodecyl sulphate (SDS)) supplemented with a protease-inhibitor cocktail (Roche; Cat. Nr. 11 836 15,300), and then sonicated. Protein concentration was determined on a NanoDropTM spectrophotometer. Proteins (~150 μg per tissue lysate) were separated on a 12% SDS-PAGE gel and then transferred on an activated (10 s in methanol, 5 min in dH2O, 10 min in Transfer Buffer (TB)) AmershamTM HybondTM PVDF membrane (GE Healthcare Life science) via the semi-dry blotting method. The membrane was subsequently blocked with 5% milk in PBS-T (0.1% Tween-20, 1 X PBS) for 1 h at RT, and incubated at 4 °C overnight with the following primary antibodies in blocking solution: rat anti-CCL2/ MCP-1 (LifeSpan BioSciences, 1:1000), rabbit anti-VCAM-1 (LifeSpan BioSciences, 1:1000), mouse anti-Tubulin (Developmental Studies Hybridoma Bank, 1:3000), rabbit anti-G-CSF (Abcam, 1:1000), and rabbit anti-GM-CSF (Abcam, 1:1000). After the membrane was washed in 1 X PBS, it was incubated for 1 h at RT with the following horseradish peroxidase (HRP)-coupled secondary antibodies in blocking solution: anti-rat HRP (Santa Cruz, 1:2000), anti-mouse HRP (Jackson ImmunoResearch, 1:3000), and anti-rabbit HRP (Jackson ImmunoResearch, 1:3000). Membranes were developed using the AmershamTM ECLTM Prime WB Detection Reagent (GE Healthcare Life science).

Quantitative analysis of the immunoblot results was assessed via band intensity measured with ImageJ (Version 1.53c, Wayne Rasband, National Institutes of Health, Bethesda, MD, USA, http://imagej.nih.gov/ij). For the densitometric analysis, β-Tubulin was used as loading control, and density levels of proteins of interest from the Cx32 KO and Cx47 KO EAE groups were normalized to the WT EAE group whose value was set at 1. Data are presented as mean ± standard deviation (SD).

### 4.5. RNA Extraction and Reverse Transcription PCR (RT-qPCR)

Total RNA was extracted from the lumbar spinal cord segment with RNeasy Lipid Tissue Mini kit (QIAGEN) following the manufacturer’s protocol and quantified with a NanoDropTM spectrophotometer. To confirm that the RNA samples are devoid of any residual contaminating genomic DNA (gDNA), standard PCR for *β-actin* (*β-actin*-F, 5′-ATCTGGCACCACACCTTCTACAATGAGCTGCG-3′; *β-actin*-R, 5′-CGTCATACTCCTGCTTGCTGATCCACATCTGC-3′), followed by 1% agarose gel electrophoresis was carried out. The absence of a *β-actin* band indicated samples were DNA-free. Aliquots of 500 ng of total RNA from each sample were reverse transcribed into cDNA with TaqMan^®^ Reverse Transcription Reagents (Applied Biosystems), following the manufacturer’s protocol.

For the RT-qPCR, a reaction volume of 20 μL per well was prepared containing 25 ng cDNA, 2 X TaqMan^®^ Universal PCR Master Mix (Applied Biosystems), and the desired 1 X TaqMan^®^ Gene Expression Assay: *Ccl2* (Mm00441242_m1), *Vcam-1* (Mm01320970_m1), and *Gapdh* (Mm99999915_g1). To determine the relative expression of the gene of interest (goi), the goi fold change was calculated utilizing the 2^−ΔΔCt^ method (Schmittgen and Livak, 2008). Each cDNA sample was tested in triplicates, both for the goi and the internal control gene (*Gapdh*, housekeeping gene). By averaging the values of the goi triplicates and of the *Gapdh* triplicates, the mean cycle threshold (Ct) value for the goi and the *Gapdh* were calculated, respectively. The mean Ct value for the goi was normalized to the respective mean Ct value for the *Gapdh* internal control using the ΔCt formula. The ΔCt values for each group (WT EAE, Cx32 KO EAE, and Cx47 KO EAE) were averaged to calculate the mean ΔCt for each group separately. From these three groups, the WT EAE group acted as the experimental control and the calibrator. The mean ΔCt values of the test groups (Cx32 KO EAE, and Cx47 KO EAE) were each normalized to the mean ΔCt of the calibrator group using the ΔΔCt formula. ΔΔCt values were transformed into fold-change values using the 2^−ΔΔCt^ formula. Data are presented as mean fold-change ± standard deviation (SD).

### 4.6. Statistical Analyses

GraphPad Prism software (Version 5.01, GraphPad Software, San Diego, CA, USA, www.graphpad.com) was used for all data analyses and presentation. To assess differences in clinical scores among the three EAE genotypes (WT EAE, Cx32 KO EAE, and Cx47 KO EAE) regular (no pairing) two-way ANOVA with post hoc Bonferroni test was used. When testing for significance in the day of EAE onset, cytokine expression, and number of cell infiltrates among the three EAE genotypes, regular (no pairing) one-way analysis of variance (ANOVA) with post hoc Tukey’s honestly significant difference (HSD) test was applied. Differences in Cx43 and GFAP intensity between the two EAE genotypes (WT and Cx47 KO EAE) were examined with unpaired two-tailed Student’s *t*-test. When the two EAE genotypes (WT and Cx47 KO EAE) were tested for their glio-vascular integrity, regular (no pairing) two-way ANOVA with post hoc Bonferroni test was carried out. Data are presented as mean ± SD and statistically significant was considered any value of *p* < 0.05.

## 5. Conclusions

In summary, Cx47-deficient mice are prone to a more severe BSCB disruption under inflammatory conditions, more pronounced astrogliosis, greater infiltration of T-cells, alongside with higher infiltration-promoting *Vcam-1* expression before EAE manifestation. The early instigator responsible for their exacerbated EAE severity appears to be their weaker BSCB; however, this has not been clearly elucidated and requires further experimentation. Since loss of Cx47 has been found in MS brain [15], and predisposes to earlier EAE onset and more severe EAE progression, further studies may lead to the development of therapeutic interventions or prophylactic treatments for MS.

## Data Availability

Data is contained within the article.

## References

[1] Wallin M.T., Culpepper W.J., Nichols E., Bhutta Z.A., Gebrehiwot T.T., Hay S.I., Khalil I.A., Krohn K.J., Liang X., Naghavi M. (2019). Global, regional, and national burden of multiple sclerosis 1990–2016: A systematic analysis for the Global Burden of Disease Study 2016. Lancet Neurol..

[2] Robinson A.P., Harp C.T., Noronha A., Miller S.D. (2014). The experimental autoimmune encephalomyelitis (EAE) model of MS: Utility for understanding disease pathophysiology and treatment. Handb. Clin. Neurol..

[3] Simmons S.B., Pierson E.R., Lee S.Y., Goverman J.M. (2013). Modeling the heterogeneity of multiple sclerosis in animals. Trends Immunol..

[4] Constantinescu C.S., Farooqi N., O’Brien K., Gran B. (2011). Experimental autoimmune encephalomyelitis (EAE) as a model for multiple sclerosis (MS). Br. J. Pharmacol..

[5] Mei F., Lehmann-Horn K., Shen Y.A., Rankin K.A., Stebbins K.J., Lorrain D.S., Pekarek K., Sagan S.A., Xiao L., Teuscher C. (2016). Accelerated remyelination during inflammatory demyelination prevents axonal loss and improves functional recovery. eLife.

[6] Yandamuri S.S., Lane T.E. (2016). Imaging Axonal Degeneration and Repair in Preclinical Animal Models of Multiple Sclerosis. Front. Immunol..

[7] Ortiz G.G., Pacheco-Moises F.P., Macias-Islas M.A., Flores-Alvarado L.J., Mireles-Ramirez M.A., Gonzalez-Renovato E.D., Hernandez-Navarro V.E., Sanchez-Lopez A.L., Alatorre-Jimenez M.A. (2014). Role of the blood-brain barrier in multiple sclerosis. Arch. Med. Res..

[8] Floris S., Blezer E.L., Schreibelt G., Dopp E., Van der Pol S.M., Schadee-Eestermans I.L., Nicolay K., Dijkstra C.D., De Vries H.E. (2004). Blood-brain barrier permeability and monocyte infiltration in experimental allergic encephalomyelitis: A quantitative MRI study. Brain.

[9] Maglione M., Tress O., Haas B., Karram K., Trotter J., Willecke K., Kettenmann H. (2010). Oligodendrocytes in mouse corpus callosum are coupled via gap junction channels formed by connexin47 and connexin32. Glia.

[10] Kim M.S., Gloor G.B., Bai D. (2013). The distribution and functional properties of Pelizaeus-Merzbacher-like disease-linked Cx47 mutations on Cx47/Cx47 homotypic and Cx47/Cx43 heterotypic gap junctions. Biochem. J..

[11] Kamasawa N., Sik A., Morita M., Yasumura T., Davidson K.G., Nagy J.I., Rash J.E. (2005). Connexin-47 and connexin-32 in gap junctions of oligodendrocyte somata, myelin sheaths, paranodal loops and Schmidt-Lanterman incisures: Implications for ionic homeostasis and potassium siphoning. Neuroscience.

[12] Kleopa K.A. (2011). The role of gap junctions in Charcot-Marie-Tooth disease. J. Neurosci..

[13] Wasseff S.K., Scherer S.S. (2011). Cx32 and Cx47 mediate oligodendrocyte:astrocyte and oligodendrocyte:oligodendrocyte gap junction coupling. Neurobiol. Dis..

[14] Srinivas M., Verselis V.K., White T.W. (2018). Human diseases associated with connexin mutations. Biochim. Biophys. Acta.

[15] Markoullis K., Sargiannidou I., Schiza N., Hadjisavvas A., Roncaroli F., Reynolds R., Kleopa K.A. (2012). Gap junction pathology in multiple sclerosis lesions and normal-appearing white matter. Acta Neuropathol..

[16] Menichella D.M., Goodenough D.A., Sirkowski E., Scherer S.S., Paul D.L. (2003). Connexins are critical for normal myelination in the CNS. J. Neurosci..

[17] Nelles E., Butzler C., Jung D., Temme A., Gabriel H.D., Dahl U., Traub O., Stumpel F., Jungermann K., Zielasek J. (1996). Defective propagation of signals generated by sympathetic nerve stimulation in the liver of connexin32-deficient mice. Proc. Natl. Acad. Sci. USA.

[18] Papaneophytou C.P., Georgiou E., Karaiskos C., Sargiannidou I., Markoullis K., Freidin M.M., Abrams C.K., Kleopa K.A. (2018). Regulatory role of oligodendrocyte gap junctions in inflammatory demyelination. Glia.

[19] Gibson-Corley K.N., Boyden A.W., Leidinger M.R., Lambertz A.M., Ofori-Amanfo G.P., Naumann W., Goeken J.A., Karandikar N.J. (2016). A method for histopathological study of the multifocal nature of spinal cord lesions in murine experimental autoimmune encephalomyelitis. PeerJ.

[20] Caravagna C., Jaouen A., Desplat-Jego S., Fenrich K.K., Bergot E., Luche H., Grenot P., Rougon G., Malissen M., Debarbieux F. (2018). Diversity of innate immune cell subsets across spatial and temporal scales in an EAE mouse model. Sci. Rep..

[21] Argaw A.T., Gurfein B.T., Zhang Y., Zameer A., John G.R. (2009). VEGF-mediated disruption of endothelial CLN-5 promotes blood-brain barrier breakdown. Proc. Natl. Acad. Sci. USA.

[22] Nitta T., Hata M., Gotoh S., Seo Y., Sasaki H., Hashimoto N., Furuse M., Tsukita S. (2003). Size-selective loosening of the blood-brain barrier in claudin-5-deficient mice. J. Cell Biol..

[23] Horng S., Therattil A., Moyon S., Gordon A., Kim K., Argaw A.T., Hara Y., Mariani J.N., Sawai S., Flodby P. (2017). Astrocytic tight junctions control inflammatory CNS lesion pathogenesis. J. Clin. Investig..

[24] Mora P., Hollier P.L., Guimbal S., Abelanet A., Diop A., Cornuault L., Couffinhal T., Horng S., Gadeau A.P., Renault M.A. (2020). Blood-brain barrier genetic disruption leads to protective barrier formation at the Glia Limitans. PLoS Biol..

[25] Konietzko U., Muller C.M. (1994). Astrocytic dye coupling in rat hippocampus: Topography, developmental onset, and modulation by protein kinase C. Hippocampus.

[26] Brand-Schieber E., Werner P., Iacobas D.A., Iacobas S., Beelitz M., Lowery S.L., Spray D.C., Scemes E. (2005). Connexin43, the major gap junction protein of astrocytes, is down-regulated in inflamed white matter in an animal model of multiple sclerosis. J. Neurosci. Res..

[27] Simard M., Arcuino G., Takano T., Liu Q.S., Nedergaard M. (2003). Signaling at the Gliovascular Interface. J. Neurosci..

[28] Miyamoto Y., Torii T., Tanoue A., Yamauchi J. (2016). VCAM1 acts in parallel with CD69 and is required for the initiation of oligodendrocyte myelination. Nat. Commun..

[29] Carlos T.M., Schwartz B.R., Kovach N.L., Yee E., Rosa M., Osborn L., Chi-Rosso G., Newman B., Lobb R., Rosso M. (1990). Vascular cell adhesion molecule-1 mediates lymphocyte adherence to cytokine-activated cultured human endothelial cells. Blood.

[30] Gimenez M.A., Sim J.E., Russell J.H. (2004). TNFR1-dependent VCAM-1 expression by astrocytes exposes the CNS to destructive inflammation. J. Neuroimmunol..

[31] Baron J.L., Madri J.A., Ruddle N.H., Hashim G., Janeway C.A. (1993). Surface expression of alpha 4 integrin by CD4 T cells is required for their entry into brain parenchyma. J. Exp. Med..

[32] Peterson J.W., Bo L., Mork S., Chang A., Ransohoff R.M., Trapp B.D. (2002). VCAM-1-positive microglia target oligodendrocytes at the border of multiple sclerosis lesions. J. Neuropathol. Exp. Neurol..

[33] Mitroulis I., Alexaki V.I., Kourtzelis I., Ziogas A., Hajishengallis G., Chavakis T. (2015). Leukocyte integrins: Role in leukocyte recruitment and as therapeutic targets in inflammatory disease. Pharmacol. Ther..

[34] Engelhardt B., Laschinger M., Schulz M., Samulowitz U., Vestweber D., Hoch G. (1998). The development of experimental autoimmune encephalomyelitis in the mouse requires alpha4-integrin but not alpha4beta7-integrin. J. Clin. Investig..

[35] Garton K.J., Gough P.J., Philalay J., Wille P.T., Blobel C.P., Whitehead R.H., Dempsey P.J., Raines E.W. (2003). Stimulated shedding of vascular cell adhesion molecule 1 (VCAM-1) is mediated by tumor necrosis factor-alpha-converting enzyme (ADAM 17). J. Biol. Chem..

[36] Singh R.J., Mason J.C., Lidington E.A., Edwards D.R., Nuttall R.K., Khokha R., Knauper V., Murphy G., Gavrilovic J. (2005). Cytokine stimulated vascular cell adhesion molecule-1 (VCAM-1) ectodomain release is regulated by TIMP-3. Cardiovasc. Res..

[37] Cook-Mills J.M., Marchese M.E., Abdala-Valencia H. (2011). Vascular cell adhesion molecule-1 expression and signaling during disease: Regulation by reactive oxygen species and antioxidants. Antioxid. Redox Signal..

[38] Ueki S., Kihara J., Kato H., Ito W., Takeda M., Kobayashi Y., Kayaba H., Chihara J. (2009). Soluble vascular cell adhesion molecule-1 induces human eosinophil migration. Allergy.

[39] Tchalla A.E., Wellenius G.A., Travison T.G., Gagnon M., Iloputaife I., Dantoine T., Sorond F.A., Lipsitz L.A. (2015). Circulating vascular cell adhesion molecule-1 is associated with cerebral blood flow dysregulation, mobility impairment, and falls in older adults. Hypertension.

[40] Fife B.T., Huffnagle G.B., Kuziel W.A., Karpus W.J. (2000). CC chemokine receptor 2 is critical for induction of experimental autoimmune encephalomyelitis. J. Exp. Med..

[41] Kennedy K.J., Strieter R.M., Kunkel S.L., Lukacs N.W., Karpus W.J. (1998). Acute and relapsing experimental autoimmune encephalomyelitis are regulated by differential expression of the CC chemokines macrophage inflammatory protein-1alpha and monocyte chemotactic protein-1. J. Neuroimmunol..

[42] Paul D., Ge S., Lemire Y., Jellison E.R., Serwanski D.R., Ruddle N.H., Pachter J.S. (2014). Cell-selective knockout and 3D confocal image analysis reveals separate roles for astrocyte-and endothelial-derived CCL2 in neuroinflammation. J. Neuroinflamm..

[43] Mahad D., Callahan M.K., Williams K.A., Ubogu E.E., Kivisakk P., Tucky B., Kidd G., Kingsbury G.A., Chang A., Fox R.J. (2006). Modulating CCR2 and CCL2 at the blood-brain barrier: Relevance for multiple sclerosis pathogenesis. Brain.

[44] Zhong L., Simard M.J., Huot J. (2018). Endothelial microRNAs regulating the NF-kappaB pathway and cell adhesion molecules during inflammation. FASEB J..

[45] Khyzha N., Khor M., DiStefano P.V., Wang L., Matic L., Hedin U., Wilson M.D., Maegdefessel L., Fish J.E. (2019). Regulation of CCL2 expression in human vascular endothelial cells by a neighboring divergently transcribed long noncoding RNA. Proc. Natl. Acad. Sci. USA.

[46] Duncker P.C., Stoolman J.S., Huber A.K., Segal B.M. (2018). GM-CSF Promotes Chronic Disability in Experimental Autoimmune Encephalomyelitis by Altering the Composition of Central Nervous System-Infiltrating Cells, but Is Dispensable for Disease Induction. J. Immunol..

[47] Ponomarev E.D., Shriver L.P., Maresz K., Pedras-Vasconcelos J., Verthelyi D., Dittel B.N. (2007). GM-CSF production by autoreactive T cells is required for the activation of microglial cells and the onset of experimental autoimmune encephalomyelitis. J. Immunol..

[48] Rumble J.M., Huber A.K., Krishnamoorthy G., Srinivasan A., Giles D.A., Zhang X., Wang L., Segal B.M. (2015). Neutrophil-related factors as biomarkers in EAE and MS. J. Exp. Med..

[49] Peng W. (2017). G-CSF treatment promotes apoptosis of autoreactive T cells to restrict the inflammatory cascade and accelerate recovery in experimental allergic encephalomyelitis. Exp. Neurol..

[50] Diederich K., Sevimli S., Dorr H., Kosters E., Hoppen M., Lewejohann L., Klocke R., Minnerup J., Knecht S., Nikol S. (2009). The role of granulocyte-colony stimulating factor (G-CSF) in the healthy brain: A characterization of G-CSF-deficient mice. J. Neurosci..

[51] Schneider A., Kruger C., Steigleder T., Weber D., Pitzer C., Laage R., Aronowski J., Maurer M.H., Gassler N., Mier W. (2005). The hematopoietic factor G-CSF is a neuronal ligand that counteracts programmed cell death and drives neurogenesis. J. Clin. Investig..

[52] Aloisi F., Carè A., Borsellino G., Gallo P., Rosa S., Bassani A., Cabibbo A., Testa U., Levi G., Peschle C. (1992). Production of hemolymphopoietic cytokines (IL-6, IL-8, colony-stimulating factors) by normal human astrocytes in response to IL-1 beta and tumor necrosis factor-alpha. J. Immunol..

[53] Zhao L.R., Navalitloha Y., Singhal S., Mehta J., Piao C.S., Guo W.P., Kessler J.A., Groothuis D.R. (2007). Hematopoietic growth factors pass through the blood-brain barrier in intact rats. Exp. Neurol..

[54] D’Amelio F.E., Smith M.E., Eng L.F. (1990). Sequence of tissue responses in the early stages of experimental allergic encephalomyelitis (EAE): Immunohistochemical, light microscopic, and ultrastructural observations in the spinal cord. Glia.

[55] Eng L.F., D’Amelio F.E., Smith M.E. (1989). Dissociation of GFAP intermediate filaments in EAE: Observations in the lumbar spinal cord. Glia.

[56] Fletcher J.M., Lalor S.J., Sweeney C.M., Tubridy N., Mills K.H. (2010). T cells in multiple sclerosis and experimental autoimmune encephalomyelitis. Clin. Exp. Immunol..

[57] Brown L.S., Foster C.G., Courtney J.M., King N.E., Howells D.W., Sutherland B.A. (2019). Pericytes and Neurovascular Function in the Healthy and Diseased Brain. Front. Cell Neurosci..

[58] Kloc M., Kubiak J.Z., Li X.C., Ghobrial R.M. (2015). Pericytes, microvasular dysfunction, and chronic rejection. Transplantation.

[59] Duz B., Oztas E., Erginay T., Erdogan E., Gonul E. (2007). The effect of moderate hypothermia in acute ischemic stroke on pericyte migration: An ultrastructural study. Cryobiology.

[60] Dore-Duffy P., Owen C., Balabanov R., Murphy S., Beaumont T., Rafols J.A. (2000). Pericyte migration from the vascular wall in response to traumatic brain injury. Microvasc. Res..

[61] Markoullis K., Sargiannidou I., Gardner C., Hadjisavvas A., Reynolds R., Kleopa K.A. (2012). Disruption of oligodendrocyte gap junctions in experimental autoimmune encephalomyelitis. Glia.

[62] Watanabe M., Masaki K., Yamasaki R., Kawanokuchi J., Takeuchi H., Matsushita T., Suzumura A., Kira J.I. (2016). Th1 cells downregulate connexin 43 gap junctions in astrocytes via microglial activation. Sci. Rep..

[63] De Pina-Benabou M.H., Szostak V., Kyrozis A., Rempe D., Uziel D., Urban-Maldonado M., Benabou S., Spray D.C., Federoff H.J., Stanton P.K. (2005). Blockade of gap junctions in vivo provides neuroprotection after perinatal global ischemia. Stroke.

[64] Frantseva M.V., Kokarovtseva L., Naus C.G., Carlen P.L., MacFabe D., Perez J.L. (2002). Velazquez Specific gap junctions enhance the neuronal vulnerability to brain traumatic injury. J. Neurosci..

[65] Tress O., Maglione M., Zlomuzica A., May D., Dicke N., Degen J., Dere E., Kettenmann H., Hartmann D., Willecke K. (2011). Pathologic and phenotypic alterations in a mouse expressing a connexin47 missense mutation that causes Pelizaeus-Merzbacher-like disease in humans. PLoS Genet..

[66] Odermatt B., Wellershaus K., Wallraff A., Seifert G., Degen J., Euwens C., Fuss B., Bussow H., Schilling K., Steinhauser C. (2003). Connexin 47 (Cx47)-deficient mice with enhanced green fluorescent protein reporter gene reveal predominant oligodendrocytic expression of Cx47 and display vacuolized myelin in the CNS. J. Neurosci..

[67] Seo J.H., Maki T., Maeda M., Miyamoto N., Liang A.C., Hayakawa K., Pham L.D., Suwa F., Taguchi A., Matsuyama T. (2014). Oligodendrocyte precursor cells support blood-brain barrier integrity via TGF-beta signaling. PLoS ONE.

[68] Niu J., Li T., Yi C., Huang N., Koulakoff A., Weng C., Li C., Zhao C.J., Giaume C., Xiao L. (2016). Connexin-based channels contribute to metabolic pathways in the oligodendroglial lineage. J. Cell Sci..

[69] Zhao Y., Yamasaki R., Yamaguchi H., Nagata S., Une H., Cui Y., Masaki K., Nakamuta Y., Iinuma K., Watanabe M. (2020). Oligodendroglial connexin 47 regulates neuroinflammation upon autoimmune demyelination in a novel mouse model of multiple sclerosis. Proc. Natl. Acad. Sci. USA.

[70] Meens M.J., Kutkut I., Rochemont V., Dubrot J., Kaladji F.R., Sabine A., Lyons O., Hendrikx S., Bernier-Latmani J., Kiefer F. (2017). Cx47 fine-tunes the handling of serum lipids but is dispensable for lymphatic vascular function. PLoS ONE.

[71] Munger S.J., Davis M.J., Simon A.M. (2017). Defective lymphatic valve development and chylothorax in mice with a lymphatic-specific deletion of Connexin43. Dev. Biol..

[72] Jones M.V., Nguyen T.T., Deboy C.A., Griffin J.W., Whartenby K.A., Kerr D.A., Calabresi P.A. (2008). Behavioral and pathological outcomes in MOG 35–55 experimental autoimmune encephalomyelitis. J. Neuroimmunol..

